# Chemokine Binding to Tenascin-C Influences Chemokine-Induced Immune Cell Migration

**DOI:** 10.3390/ijms241914694

**Published:** 2023-09-28

**Authors:** Alissa Domaingo, Philipp Jokesch, Alexandra Schweiger, Martha Gschwandtner, Tanja Gerlza, Manuel Koch, Kim S. Midwood, Andreas J. Kungl

**Affiliations:** 1 Institute of Pharmaceutical Sciences, Karl-Franzens-University Graz, Schubertstr. 1, 8010 Graz, Austria; 2 Kennedy Institute of Rheumatology, University of Oxford, Roosevelt Drive, Oxford OX3 7FY, UK; 3 Institute for Dental Research and Oral Musculoskeletal Biology, Faculty of Medicine and University Hospital Cologne, University of Cologne, Joseph-Stelzmann-Str. 52, 50931 Cologne, Germany; 4 Antagonis Biotherapeutics GmbH, Strasserhofweg 77a, 8045 Graz, Austria

**Keywords:** extracellular matrix, tenascin-C, chemokines, glycosaminoglycans, fluorescence, circular dichroism

## Abstract

Tenascin-C (TNC) is a complex glycoprotein of the extracellular matrix (ECM) involved in a plethora of (patho-)physiological processes, such as oncogenesis and inflammation. Since chemokines play an essential role in both disease processes, we have investigated here the binding of TNC to some of the key chemokines, namely CCL2, CCL26, CXCL8, CXCL10, and CXCL12. Thereby, a differential chemokine-TNC binding pattern was observed, with CCL26 exhibiting the highest and CCL2 the lowest affinity for TNC. Heparan sulfate (HS), another member of the ECM, proved to be a similarly high-affinity ligand of TNC, with a Kd value of 730 nM. Chemokines use glycosa-minoglycans such as HS as co-receptors to induce immune cell migration. Therefore, we assumed an influence of TNC on immune cell chemotaxis due to co-localization within the ECM. CCL26- and CCL2-induced mobilization experiments of eosinophils and monocytes, respectively, were thus performed in the presence and the absence of TNC. Pre-incubation of the immune cells with TNC resulted in a 3.5-fold increase of CCL26-induced eosinophil chemotaxis, whereas a 1.3-fold de-crease in chemotaxis was observed when monocytes were pre-incubated with CCL2. As both chemokines have similar HS binding but different TNC binding affinities, we speculate that TNC acts as an attenuator in monocyte and as an amplifier in eosinophil mobilization by impeding CCL2 from binding to HS on the one hand, and by reinforcing CCL26 to bind to HS on the other hand.

## 1. Introduction

The extracellular matrix (ECM) is a complex, three-dimensional network of different matrix molecules, including fibrous proteins, glycoproteins, proteoglycans, glycosaminoglycans (GAGs), enzymes, and ECM receptors [1]. It is actively involved in tissue morphogenesis, differentiation, and homeostasis by acting as a physical scaffold for cellular components and providing biochemical and biomechanical signals [2]. In other words, the ECM is an interconnecting and dynamically organized assembly of several macromolecules providing a mechanical scaffold for embedded cells and further participating in various (patho-)physiological cellular processes. Its structural and functional variety in every organ and tissue is a result of different cell types forming the ECM and offering a wide range of regulations and modifications, especially in diseases like inflammation and cancer. In fact, pathological conditions can either cause or be a result of ECM abnormalities [3,4].

Remodeling of the ECM occurs during tumor progression. It is mediated by enzymes and by the secretion of tumor-specific ECM molecules that reorganize the ECM, which is stiffened by lipoxygenase and transglutaminase [2]. ECM composition plays an important role in tumor progression, and proteomics-based studies have revealed precisely how the ECM changes in various cancerous tissues [5,6]. For instance, the expression pattern of tenascin-C is significantly different [7,8]. It is often described as an oligomeric glycoprotein (180–300 kDa) of six monomers building a hexabrachion, with each of them containing four distinct domains [9,10]. However, it remains to be elucidated how many of the putative glycosylation sites are involved and if TNC occurs as a proteoglycan [11].

Nevertheless, the ability of TNC to interact with a wide range of molecules makes it an important member in several biochemical processes such as cell spreading, migration, survival, growth, and adhesion [12]. These properties are a result of the fact that TNC underlies various (post-)transcriptional regulations and post-translational modifications [13]. Therefore, several isoforms and conformations with different functions have been described already. Above all, a variety of glycosylation patterns are likely to play a major role in altering cell behavior [9,11]. A clear and well-defined mode of action in physiological and pathological processes has not yet been associated with TNC.

One of the known interaction partners of matrix molecules are extracellular ligands such as chemotactic cytokines [3]. These chemokines are small (7–15 kDa) secreted, basic proteins, which are essential in directing different leukocytes from the lumen of blood vessels into the side of adjacent inflamed tissue through a local concentration gradient [14,15]. However, in vivo, chemokines do not act alone. The chemotactic function requires an interaction complex of chemokine, chemokine-specific G-protein coupled receptor (GPCR), and- glycosaminoglycans (GAG) as co-receptors [15,16]. For example, the monocyte chemoattractant protein-1, briefly MCP-1 or CCL2, specifically attracts monocytes and some other immune cells by binding their GPCR CCR2 on the cell surface. As a proinflammatory chemokine, CCL2 is known for its upregulation in various inflammatory diseases, including rheumatoid arthritis, atherosclerosis, and multiple sclerosis, and furthermore, it is involved in tumor progression [17,18,19,20]. According to the literature, this variety can be ascribed to the extended functions of CCL2 beyond its chemotactic properties [21]. Moreover, the specific relation between TNC and CCL2 is also described, as both proteins are involved and highly overexpressed in the case of breast cancer [12,20,22,23,24]. In fact, TNC might cause an elevated CCL2 expression level [25,26].

CCL26, also called eotaxin-3, is a pro-inflammatory chemokine that interacts exclusively with the CCR3 receptor on the eosinophil surface and is a major cause of eosinophilia-related diseases [27]. It belongs to the eotaxin family, which consists of three members: CCL11 (eotaxin-1), CCL24 (eotaxin-2), and CCL26 (eotaxin-3) [28]. Due to their unique ability to stimulate eosinophil chemotaxis via CCR3 activation, the three proteins have been classified as the eotaxin family, although they share sequence homologies of less than 40% [29]. CXCL8 (CXC motif ligand 8, or IL-8) is the major neutrophil chemoattractant in humans, and its levels correlate with lung disease severity in sputum and BAL [30]. Multiple sclerosis, COPD, psoriasis, diabetes, encephalitis, rheumatoid arthritis, asthma, cystic fibrosis, and many other diseases have also been associated with elevated CXCL8 levels [31,32,33,34]. SDF-1α, or CXCL12, is a small (8 kDa) soluble chemoattractant cytokine that binds and activates the seven-span transmembrane G-protein coupled receptor (GPCR) CXCR4.

SDF-1α acts as an attractant for lymphocytes, dendritic cells, hematopoietic stem cells, endothelial cells, and progenitor cells [35,36,37,38,39]. Several studies have shown that it induces chemotaxis of monocytes, macrophages, and mesenchymal stem cells [40,41,42]. TNC is highly expressed in tumors, and during chronic inflammation and bacterial or viral infection, marking it as a potential factor in the control of the immune system. By binding TNC to CXCL12, it was recently shown that tumor-infiltrating leukocytes are trapped in the stroma, leading to tumor surveillance. When blocking the respective GPCR receptor CXCR4, a greater number of macrophages and CD8+ T-cells infiltrate the tumor, reducing tumor growth and metastasis [43,44]. Additionally, soluble factors, including cytokines, are already described as transcriptional regulators of TNC expression [26]. Thus, a cross-interaction between TNC and chemokines, in general, is assumed.

It has already been mentioned that GAGs—particularly, among them, the ubiquitously located heparan sulfate (HS)—act as co-receptors in chemotactic signaling; they participate in anchoring, presentation, and conformational activation of chemokines and are required for in vivo function [16]. GAGs are linear polysaccharides consisting of beta-D-glucuronic acid and alpha-d-glucosamine building blocks, which are massively modified by various sulfations and acetylations, making them substantially negatively charged and therefore highly suitable for the binding of basic proteins such as the chemokines. Among all GAGs, HS exhibits the largest amount of structural heterogeneity, reflecting tissue and disease-specific modification patterns that are added during and after synthesis in the Golgi apparatus [45]. In vivo, HS exerts its biological and pathological functions as covalently *O*-linked glycanation parts of certain core proteins, the so-called HS-proteoglycans (HSPGs) [46]. In particular, HSPGs are known for their essential role in chemotactic processes, which has been demonstrated for the CCL2-CCR2 axis [47]. Furthermore, it has been shown that the integration of TNC into the ECM depends on the availability of HSPGs [48]. In addition to their manifold biologic functions, HSPGs, such as the membrane-bound glypicans (GPC) and syndecans (SDC), often serve as the initial anchor point for many pathogens including viruses, which enables the subsequent interaction of viral proteins with their specific protein receptors on the host cell, making HSPGs co-receptors also for viral cell infection (Refs). Finally, it has been observed that GAGs can act as molecular chaperones for certain proteins by preventing aggregation and thus supporting protein folding [49,50,51].

We have recently shown that GAGs present on target immune cells contribute to chemotaxis, in addition to GAGs located at the site of vessels where immune cells extravasate into affected tissues [52,53]. Chemokine secretion, immobilization, and receptor activation are apparently mediated by GAGs situated on both sites of the immunological reaction, that is, on the epithelial/endothelial vessel cells as well as on the blood-resident immune cells. This concerted action of GAGs might contribute to overcoming in vivo the promiscuity observed in vitro for most chemokine-chemokine receptor pairs, which renders many chemokine receptors responsive to more than one chemokine and allows one chemokine to activate more than one receptor [54].

Whether the interaction of GAGs with certain chemokines is specific, that is, whether the biological effect induced by this molecular encounter depends upon the mutual sequence pattern-specificity and non-covalent bonding complementarity between a GAG ligand and its chemokine interaction partner, is an ongoing discussion within the GAG community [55]. Since the affinities between GAGs and proteins are typically not very high (ranging in the low micromolar and high nanomolar Kd values), it is very important to know if the interaction is sequence pattern specific in order to infer a certain structure-function relationship. Several unique GAG ligands have been identified for various GAG-binding proteins, among them some chemokines [56,57,58,59,60]. Bi-molecular interactions are, however, a very rare event in vivo since biomolecules tend to exert multiple interactions with different partners at the same time, which implies a regulation of the underlying biological function on top of the simple bi-molecular binding. From this viewpoint, multiple simultaneous interactions in the ECM should be seen.

Therefore, the present paper addresses the question of whether and how strong TNC interacts with HS, the proto-typical GAG found in the ECM, as well as with chemokines, and whether these interactions have structural and functional consequences, making them potential molecular interfaces for therapeutic interventions.

## 2. Results

### 2.1. Far-UV Circular Dichroism Spectroscopy

**Figure 1:**
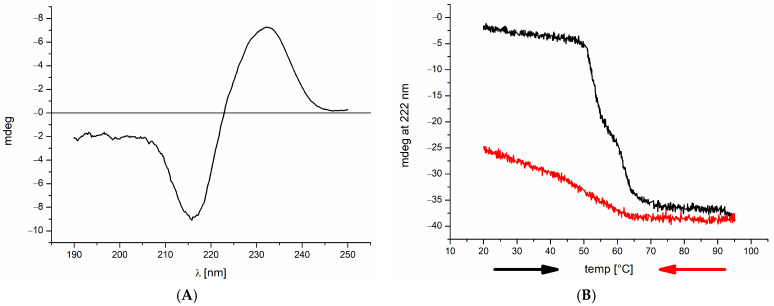
(**A**) Far-UV CD spectrum of TNC: the spectrum exhibits main bands characteristic of an all-ß-sheet protein with almost no α-helical portion; data were recorded at a concentration of 5 µM in phosphate buffer (see Materials and Methods) to avoid background absorption; the curve shown is the mean of 5 independent measurements. (**B**) Temperature-induced un- and refolding of 5 µM TNC in phosphate buffer (black and red line, respectively; arrows indicate the direction of temperature change); far-UV CD spectral response at 222 nm was recorded at every 0.2 °C temperature increment (for decrease = unfolding and increase = refolding), and the values were plotted against the respective temperature; the resulting decrease/increase in the CD signal is the result of a wavelength shift due to TNC unfolding.

Currently, there is no 3-D structure of full-length TNC available. We have therefore recorded and analyzed, as a first step, the far-UV spectrum of the protein to obtain an overall impression of the TNC structural fold (see Figure 1A). The protein exhibits large contributions of ß-sheet and (ß-)turns with almost undetectable α-helical parts (analysis with bestsel.elte.hu gave 58% antiparallel and 11% parallel sheet plus 31% of other structures). In this respect, TNC resembles typical IgG-type antibodies. The extensive (ß-)turn regions indicate a manifold of potential ligand binding sites. Upon temperature-induced unfolding, TNC switches into a non-native state between 50° and 60° (see Figure 1B), which is indicative of a relatively stable overall structure at ambient as well as at body temperature. Upon cooling down, the protein refolds spontaneously (i.e., without any stable intermediates) but only partially, which means that large parts of TNC reside most likely in a semi-stable configurational state. Such “loose” (i.e., flexible) conformational regions are again indicative of ligand binding regions, which become structurally induced and thus stabilized upon ligand interactions.

### 2.2. TNC Interaction Studies

**Figure 2:**
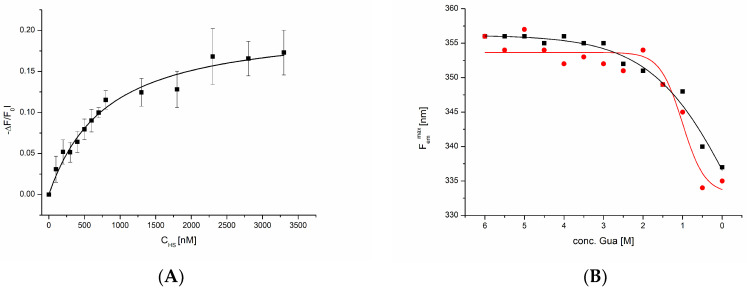
(**A**) Binding isotherm of HS interacting with TNC using the intrinsic, natural tryptophan fluorescence of TNC, which provided a strong fluorescence emission signal; the TNC input concentration was 100 nM, and HS was added in 100 nM steps (avoiding strong dilution) until saturation was reached; ligand-induced fluorescence quenching was inferred from protein emission spectra (300–400 nm) at each added ligand concentration, and the background-corrected area under the curve was related to the unquenched emission and plotted against the ligand concentration. (**B**) Fluorescence-detected chaotrope-induced re-folding of TNC in the absence (black curve) and in the presence (red curve) of HS; again, the intrinsic tryptophan fluorescence of the protein (100 nM) was used, and the hypsochromic shift of the fluorescence emission maximum induced by lowering the chaotrope concentration was indicative of protein refolding.

Such a conformationally inducing effect was observed with one of TNC’s potential ECM ligands, heparan sulfate (HS). GAGs such as HS and chondroitin sulfate (CS) represent an important class of polysaccharides that, as O-glycan components of proteoglycans, contribute to the structural and functional ECM meshwork. We have investigated whether and how strongly HS interacts with TNC and thus potentially impacts the structure and function of the protein. The binding isotherm of HS binding to TNC, as determined by isothermal fluorescence titration (IFT), is displayed in Figure 2A. From these data, a Kd value of 0.73 µM was calculated. This strong binding affinity of HS to TNC was found to also affect the refolding behavior of the protein: in chaotrope-induced refolding experiments of TNC under fluorescence detection, the protein exhibited a clear transition into a native(-like) state (point of inflexion at 1.5 M guanidine hydrochloride), which was not detected in the absence of HS (see Figure 2B).

**Figure 3:**
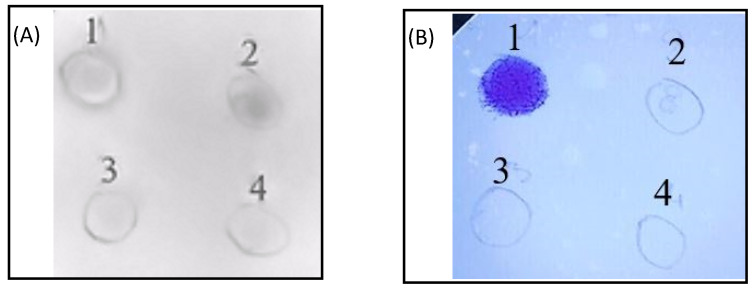
Dot blot analysis of (1) 4 µg HS in water, (2) 4 µg TNC in phosphate buffer, (3) 4 µg TNC pre-treated with 8 M urea, and (4) 4 µg TNC pre-treated with 6 M GdnCl; after 1 h incubation with chaotropes, the buffer was exchanged to phosphate buffer by serial dilution (ultrafiltration using 30 kDa cutoff filters). (**A**) displays anti-HS antibody staining; (**B**) displays azure A staining.

As HS is apparently a folding enhancer of TNC, we were interested next in whether TNC is a glycanated protein (i.e., if the protein is potentially itself a proteoglycan). For this purpose, dot blot experiments were performed using glycan detection azure A as the glycan-specific dye, as well as an HS-directed antibody. The results are displayed in Figure 3. Using the HS-directed antibody, a glycan-related signal was observed for HS in solution, as well as for TNC (Figure 3A). Interestingly, azure A staining did not give a GAG-related signal for TNC; a clear signal was detected only for HS in solution (see Figure 3B).

Upon protein denaturation with the chaotropes urea and GdnCl, antibody staining of TNC could also not be detected anymore. From these experiments—together with the above-described high affinity of TNC for HS—we conclude that HS has been co-purified with TNC, hence the positive antibody signal with the native protein, but that it is not covalently linked to the protein, since the antibody signal became lost following protein denaturation. Due to the diamine nature of azure A, the dye might not be able to detect HS when the glycan is bound to TNC (via ionic interaction with basic amino acids, such as lysine and arginine, of the protein).

Since TNC is upregulated under various inflammatory conditions, we investigated next whether certain chemokines interact with TNC [61]. Chemokines, once secreted, typically exhibit a short residual time in serum, which is significantly extended by binding to GAGs such as HS. Thereby, the site of chemokine secretion is also typically marked by the hot spot of inflammation. In this way immobilized, chemokines convey their chemotactic message via interactions with GPC receptors on target immune cells. Considering the strong binding of TNC to HS, we aimed to investigate a potentially synergizing role of TNC—and thus of the ECM—in chemotaxis. It was, therefore, interesting to see whether HS and TNC act in concert within the ECM to display chemokines to or on immune cells, thereby stimulating or attenuating the migration of target immune cells. As a first step, we have studied the binding affinities of TNC to a set of chemokines that are chemotactic for different immune cells.

**Figure 4:**
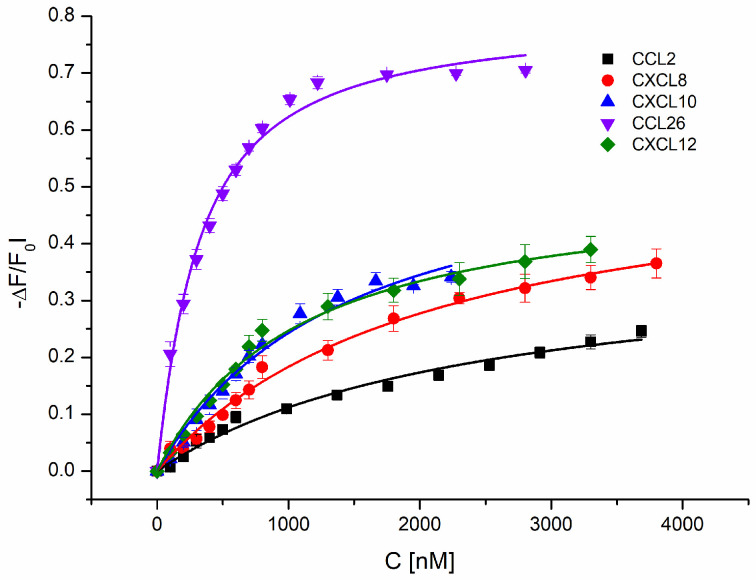
Binding isotherms of FITC-labelled TNC binding to CCL2 (Kd = 2.33 ± 0.39 μM), CXCL8 (Kd = 1.99 ± 0.2 μM), CXCL10 (Kd = 1.36 ± 0.22 μM), CXCL12 (Kd = 1.01 ± 0.10 μM), and CCL26 (Kd = 0.27 ± 0.02 μM); the TNC input concentration was 100 nM, and HS was added in 100 nM steps (avoiding strong dilution) until saturation was reached; ligand-induced fluorescence quenching was inferred from protein emission spectra (300–400 nm) at each added ligand concentration, and the background-corrected area under the curve was related to the unquenched emission and plotted against the ligand concentration.

Kd values for TNC interacting with chemokines were again determined by IFT experiments (see Figure 4). However, in order to become independent from potentially misleading overlapping fluorescence signals emitted by tryptophan residues in both interaction partners, TNC and chemokines, TNC alone was fluorescently labelled by FITC. A panel of different chemokines was then titrated to TNC—namely CCL2, CXCL8, CXCL10, CXCL12, and CCL26—with the prospective aim to correlate binding affinities with an impact on the corresponding immune cells (i.e., monocytes, neutrophils, T-cells, dendritic cells, and eosinophils, respectively). The bi-molecular saturation curves of chemokines binding to TNC are shown in Figure 4. All chemokines exhibited binding affinities with Kd values in the low micromolar range except for CCL26, for which a significantly lower Kd value of 0.27 µM was found, indicating a much higher affinity than for the other chemokines.

### 2.3. Chemotaxis

**Figure 5:**
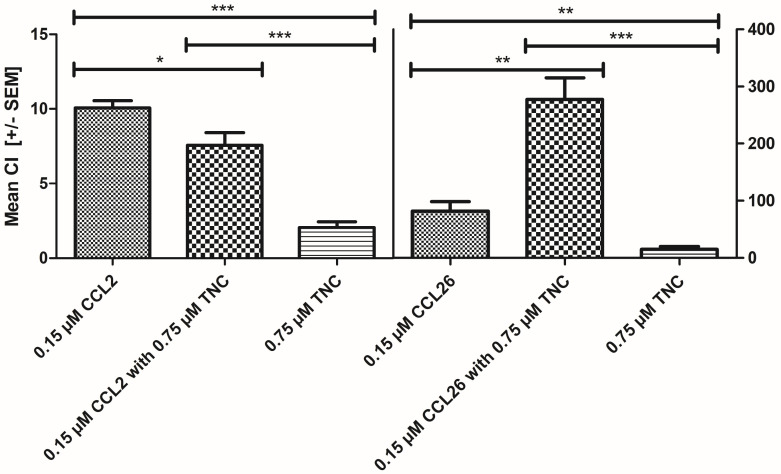
CCL2- (**left**) and CCL26-induced (**right**) chemotaxis of monocytes and eosinophils, respectively, in combination or without TNC. Data were corrected for the corresponding background migration, and data are shown as the mean chemotactic index (CI) with SEM. Statistical analysis was performed using Student’s t-test, comparing data sets with corresponding untreated chemokine concentrations. * *p* < 0.05, ** *p* < 0.01, *** *p* < 0.001 were considered as statistically significant.

Next, we investigated the influence of TNC on chemokine-mediated immune cell mobilization. For this purpose, we focused on the chemotactic effect of TNC only on the chemokines with the highest and the lowest binding affinity, that is, CCL26 and CCL2 (see Figure 5).

**Figure 6:**
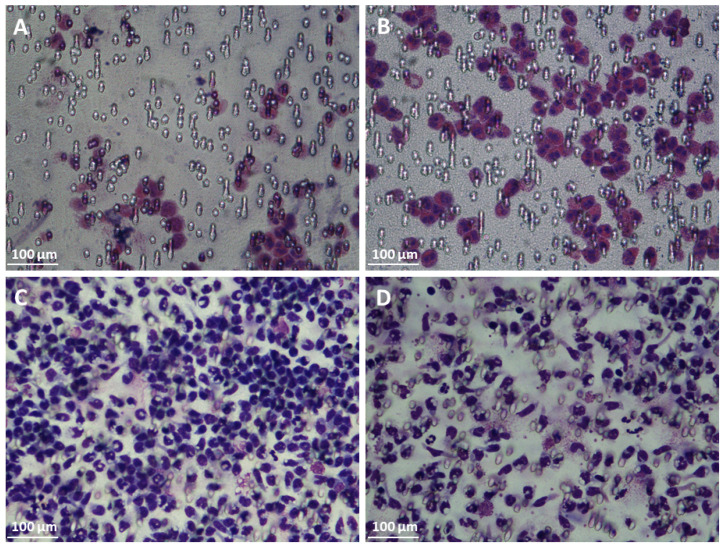
CCL26-induced chemotaxis of eosinophils without (**A**) and in the presence of TNC (**B**); and CCL2-induced chemotaxis of PBMC (Monocyte) without (**C**) and in the presence of TNC (**D**). CCL26/TNC interactions increase eosinophilic granulocyte migration, whereas CCL2/TNC complexes attenuate monocyte mobility.

Pre-incubation of chemokines with TNC resulted in a 3.5-fold amplification of CCL26-induced eosinophil chemotaxis, whereas a 1.3-fold reduction in chemotaxis was observed when monocytes were pre-incubated with CCL2 (see Figure 5 and Figure 6). As both chemokines have similar HS binding but different TNC binding affinities, we speculate that TNC acts as an attenuator in monocyte (PBMC) and as an amplifier in eosinophil mobilization by preventing CCL2 from binding to HS on the one hand and by enabling CCL26 to bind to HS on the other [62,63]. This is supported by our recent finding that HS on monocytes as well as eosinophils is important for chemotaxis [52,53]. The different TNC binding affinities of the two chemokines might thus suggest different binding sites on TNC. This will be investigated in the future by studying binding affinities to isolated TNC domains.

## 3. Discussion

The composition as well as the topology of the ECM, and thus its functions, change upon disease onset and progression, such as in cancer [64]. In general, the TNC-gene expression is tightly controlled and mainly restricted to the period of embryo development. Healthy adults show only small amounts of TNC in stem cells niches, the central nervous system, and regions exposed to high tensile stress, such as tendons [11,65]. However, during tissue remodeling (e.g., in the process of wound healing or under certain pathological conditions), the synthesis of TNC is rapidly re-induced and highly upregulated [12,66]. The TNC-gene expression is regulated by various factors [26]: transcription factors including cytokines and growth factors [66,67], matrix metalloproteinases and integrins [9], as well as mechanical stress and overload [68,69]. Next to inflammatory leukocytes, primarily stromal cells are responsible for TNC production upon tissue damage [10,12]. Upregulated TNC allows effective tissue repair by supporting inflammatory and fibrotic processes. Usually, down-regulation of TNC expression starts immediately after completing tissue repair. This feedback mechanism was found to be absent in several diseases [70]. Persistent adult de novo expression of TNC is therefore often regarded as a hallmark of chronic inflammation and cancer. The exact role of TNC during pathophysiological events, however, remains unknown. Nevertheless, a large number of diseases have been linked to the presence of altered TNC expression levels [12].

A striking example of the role of TNC in chronic inflammation is rheumatoid arthritis (RA), which is accompanied with high TNC levels in the cartilage, synovium, synovial fluid, and blood of RA patients. The current knowledge of how TNC participates in the pathology of RA is reviewed in [71]. In this case, TNC exhibits bifunctional properties as it (i) beneficially influences joint tissue repair but (ii) is also responsible for inflammatory and fibrotic reactions. These contrasting properties in RA can presumably be ascribed to the wide range of TNC post-translational modifications, such as citrullination [72].

TNC is further associated with an elevated expression level in tumor cells and a poor prognosis in several forms of cancer, such as brain tumors or breast cancer [73,74,75]. For these cases, regulatory functions of tumor- and angiogenesis, immunomodulation, as well as metastasis were ascribed to the matrix molecule by modulating the behavior of stromal and epithelial cancer cells (mainly via integrins). Generally speaking, the role of TNC in cancer includes cell proliferation, differentiation or migration, as well as the upregulation of pro-inflammatory and tumor-promoting cytokines due to activation of toll-like receptor-4 (TLR4) [75,76]. Interestingly, some of these processes are similar to those found in embryonic development and tissue repair [70,75,77].

In the above-mentioned diseases, both TNC and chemokines play essential roles in disease progression, making them potential therapeutical targets for such indications [78]. While chemokines and tenascin-C have been individually described as pivotal players in cancer, inflammation, and other pathological conditions, little is known about how their interplay affects each other’s function and thus disease progression [61,79,80,81,82]. Immune (but also tumor) cell migration was for a long time considered to be mono-dimensional (i.e., regulated and driven only by chemokines), secreted at the site of inflammation and their respective GPCRs located on the immune cells. This view was extended by the finding that most chemokines do not only interact with one receptor—thus attracting more than one type of immune cell—and that most chemokine receptors also interact with more than one chemokine (i.e., a certain type of immune cell can be recruited by more than one chemokine) [83]. This view was further extended by the discovery that glycosaminoglycans (GAGs) on immune (and cancer) cells act as co-receptors for chemokines, enabling their presentation at the site of immune cell tissue infiltration, as well as preventing proteolytic degradation of chemokines in serum, thereby extending the half-life and availability of these proteins [55].

Due to their involvement in the same diseases, we have asked ourselves whether TNC affects chemokine-induced immune cell mobilization and what role GAGs play in this scenario [84]. Understanding these interactions is of high therapeutic relevance since a better insight into the chemokine interaction network will allow for better therapeutic strategies when certain chemokines can be targeted to fight cancer and chronic inflammatory diseases (for which many clinical studies have failed in the past). In our study, we have shown that TNC is a strongly binding ligand of chemokines and thus represents a potential additionally regulating partner in chemotaxis. Kd values in the lower micromolar or high nanomolar range were found for chemokines binding to TNC (see Figure 4). In particular, CCL26 binds to tenascin C with high affinity, and when added to eosinophils, an increase in CCL26-induced chemotaxis was observed. CCL2, which was already described to be involved in breast cancer as a potent TNC binder, leads to a decrease in monocyte cell migration [20,85]. Regarding molecular recognition, both chemokines’ GPC receptors, particularly the extracellular domains of CCR2 on monocytes and CCR3 on eosinophils, exhibit low sequence homology with TNC [86]. We can therefore rule out a direct competition between chemokines binding to their respective GPCR and binding to TNC. A closer look at the amino acid sequences of CCR2 and CCR3 revealed two potential GAG/HS binding sites in CCR3, located on the second (basic cluster ^196^RHFHTLR^202^) and third (basic cluster ^275^RSKH^278^) extracellular loop, whereas no such basic cluster could be identified in CCR2 [87]. TNC, on the other hand, exhibits two potential GAG/HS binding sites: one at the N-terminus (basic cluster ^26^KKVIRHKR^33^) and one at the C-terminus (basic cluster ^2192^RNLEGRRKR^2200^). We therefore hypothesize that a simultaneous HS binding to CCR3, CCL26 and TNC leads to an agonistic/mobilizing behavior of TNC with respect to eosinophil migration. On the other hand, the lack of direct HS binding to CCR2—but presence of binding to TNC and CCL2—leads to an attenuation of monocyte migration in the presence of TNC. The axis between HS and TNC could thus be interpreted as a modulator of immune cell mobilization and migration.

## 4. Materials and Methods

### 4.1. Materials

Heparan sulfate was purchased from Celsus (Cincinnati, OH, USA), and all other chemicals from Merck (Merck, Darmstadt, Germany), if not stated otherwise. The used phosphate-buffered saline (PBS) contains 10 mM phosphate buffer and 137 mM NaCl with a pH of 7.35.

### 4.2. Preparation of Proteins

Recombinant human TNC (hTNC) and murine (mTNC) TNC were produced according to well-established protocols [11]. A sequence-based alignment of both TNCs was made using the Clustal Omega program via the UniProt.org database and displayed an identity of over 84%. Moreover, it has already been revealed that although the human and murine proteins have differences in their alternative splicing, they exhibit a very similar domain structure [11].

A collection of different recombinant chemokines of human origin was used for the binding studies with TNC. All chemokines were expressed and purified in-house as described in [62]. An adapted purification protocol was used for CXCL8 and CCL2. Harvested cells were resuspended in 4× volume 20 mM Tris, 50 mM NaCl, 1 mM EDTA, pH 8, and lysed by sonication on ice. Protein-containing inclusion bodies were separated by centrifugation (20,000× *g*, 1 h, 4 °C) and resuspended in 8 M Gua in 50 mM Tris pH 6.5 for 1 h at RT (10× original pellet volume). After centrifugation (20,000× *g*, 1 h, 4 °C), a two-step refolding process was carried out (1:5 refolding buffer 50 mM Tris, pH 7.5 + 0.1% Tween 20, 1 h at 4 °C stirring, pH lowered to 4.5, 20 min at 4 °C stirring, followed by 1:4 dilution in water, 20 min at RT stirring). After another centrifugation step, the diluted refold was loaded onto preequilibrated (50 mM Tris pH 7.5) cation exchange Fractogel EMD SO3- (Merck, Darmstadt, Germany) using a linear gradient to 50 mM Tris, 2 M NaCl pH 7.5. Protein-containing fractions were further purified using rpHPLC on a C18 column (Merck, Darmstadt, Germany) as described in [62], followed by an on-column refold using the cation exchange material SP Sepharose FF (Cytiva, Marlborough, MA, USA, as described for EMD). Fractions were concentrated using Amicon Ultracell centrifugal devices (Merck, Darmstadt, Germany). Protein content was determined by either BCA Assay or UV280. Fractions were checked for protein content after every purification step on SDS PAGE Gels stained with 0.1% Coomassie in MeOH/HAc.

### 4.3. Isothermal Fluorescence Titration (IFT)

The binding studies of TNC with different chemokines were performed using isothermal fluorescence titration (IFT). The method is able to determine binding affinities with high sensitivity and robustness by observing a decrease in the fluorescence signal. This quenching is a result of structural re-arrangement of the protein after interaction with the ligand. The higher the binding affinity, the higher the quenching. One prerequisite for performing IFT measurements is that the protein contains tryptophan (Trp) residues as intrinsic chromophores [88]. mTNC, a protein about 230 kDa in size, contains several tryptophan residues, but so do chemokines. Therefore, in order to avoid signal ambiguities, fluorescein isothiocyanate (FITC; Merck, Darmstadt, Germany) was attached as an extrinsic fluorophore to TNC via primary amines with the following procedure. The required amount of murine TNC (1 mg/mL in PBS) was incubated with a 4 molar excess of FITC (5 mg/mL in DMSO) for 2 h at room temperature. Then, desalting columns (Zeba Spin, Thermo Scientific, Waltham, MA, USA) were used to remove the abundant FITC. Quality control was performed using SDS-PAGE and Coomassie staining. While working with FITC-labeled protein, the exclusion of light is necessary, which was ensured by using tin foil.

The IFT measurements were implemented on a Fluoromax-4 Spectrofluorometer (Horiba Scientific, Kyoto, Japan) at an excitation wavelength of 495 nm over the range of 500–600 nm. Further settings were slit widths of 5 nm for excitation as well as emission, temperature of 20 °C, and scan speed of 500 nm/min. Before measurement, 100 nM FITC-labeled mTNCs (in PBS, 500 µL) were equilibrated for 30 min. Then, aliquots of ligand (different chemokines) in a concentration range from 100 to 500 nM were added, considering a dilution effect. After each titration step, the solution was mixed and equilibrated for 1 min before recording the spectra. The area under the curve of the obtained spectra was used for data analysis. Since measuring was performed at 495 nm, no background correction was necessary. The binding isotherms were generated by plotting the relative change in fluorescence intensity (−∆F/F0I) against the concentration (C) of added ligand. Here, ∆F means F − F0, where F describes the fluorescence emission at a particular ligand concentration, and F0 defines the fluorescence emission in the absence of a ligand. The mean and standard error of the mean (SEM) of three independent measurements were calculated and further analyzed in Origin 8.0 (OriginLab, Northampton, MA, USA) by a non-linear regression fit. However, neither the stoichiometry of the interaction partners nor oligomerization during measurements were considered in the data analysis because even an estimation for specific binding sites for a defined protein would be impossible. Further, the oligomerization behavior has not been understood completely [88]. In the used data fitting model, the number of binding sites was therefore set at 1, and the molecular concentrations of the proteins were considered as described.

### 4.4. Far-UV Circular Dichroism Spectroscopy (CD)

Far-UV circular dichroism was used to determine secondary structural changes during thermal un- and refolding of TNC. CD describes the variable absorption of plane polarized light, which consists of a left- and right-handed circularly polarized component. Elliptical polarization results if these two components are not equally absorbed by chiral substances. Therefore, CD refers to optical rotation [89]. Usually, spectropolarimeters are used to measure CD spectra as a function of wavelength; the differences in ellipticity, however, are reported in degrees (mdeg). The far-UV CD, for example, provides information about the secondary structure of proteins by assessing the absorption of the peptide bond in the region at and below 240 nm. The conformation of the peptide bonds determines the position and intensity of the CD bands. A typical far-UV CD spectrum exhibits a slight but wide transition around 220 nm and a stronger transition around 190 nm [90]. Proteins with α-helices only, for instance, cause a double negative band at 208 and 222 nm, as well as a more intense positive band at around 190 nm [89].

The measurement of far-UV CD spectra requires the mean residue weight (MRW) for the peptide bond of a certain protein. It is calculated (MRW = M/(N − 1) based on its molecular weight (M) and the number of amino acids (N; the number of peptide bonds: N − 1). Using far-UV CD during an increase in temperature also provides information about protein stability and changes in secondary structure as a result of denaturation [90]. The measurements of 5 µM mTNC were performed in Hellma QC (Hellma, Jena, Germany), using 1 mm cuvettes on a Jasco J-1500 CD spectropolarimeter (Jasco, Tokyo, Japan). Thermal unfolding and refolding were measured at 222 nm with a data point resolution of 0.2 nm and a response time of 1 s.

Further measurement parameters were a scan speed of 50 nm/min and a bandwidth of 1 nm. During denaturation with temperature, the spectra (190–250 nm) were collected after every 10 °C from 20–90 °C, and equally for renaturation from 90° to 20 °C. For background correction, a spectrum of 1x PBS was also taken. For primary data analysis, the software Jasco CD spectra analysis (Version 1.53.06) was used. Then, the mean residue ellipticities of five averaged measurements were plotted against the wavelength using the program Origin 8.0.

### 4.5. Fluorescence-Detected Chaotrope-Induced Refolding

To investigate the influence of HS on the refolding process of TNC, chaotrope-induced refolding experiments were conducted. Unfolded TNC was prepared in decreasing concentrations of GdnCl (MP Biomedicals LLC, Santa Ana, CA, USA), and the intrinsic tryptophan fluorescence emission was measured. For initial unfolding of TNC, a 1 µM protein solution was prepared in 8 M GdnCl and incubated at RT for 10 min. For the refolding, 100 nM TNC was prepared with decreasing GdnCl concentrations in PBS starting at 6 M GdnCl and decreasing to PBS only in 0.5 M GdnCl steps. Each sample was incubated for 5 min at RT before measurements. For each concentration step, a second sample was prepared with HS added at a 5-fold molar excess (500 nM). The fluorescence was recorded on a Fluoromax-4 Spectrofluorometer (Horiba Scientific, Kyoto, Japan) at an excitation wavelength of 280 nm. Spectra were recorded from 300 to 400 nm. Slits were set to a width of 3 nm for excitation and emission, the scan speed was 500 nm/min, and the temperature was 20 °C.

The emission maximum of each spectrum was determined and blotted against the GdnCl concentration. Using Origin 8.0, a Boltzman fit was used to calculate the point of inflexion for refolding with and without addition of HS.

### 4.6. Dot Blot Analysis

To remove GAGs from the surface of TNC, it was incubated with either urea or guanidine hydrochloride (GdnCl) followed by a DotBlot analysis. As reference, heparan sulfate (Celsus, Cincinnati, OH, USA) and untreated TNC was used. Purified TNC was incubated for 1 h with 8 M urea (Merck) and 8 M guanidine hydrochloride (GdnCl; Merck) at RT in order to break non-covalent GAG bindings. After the incubation, the free/detached GAGs, the urea, and the GdnCl were removed using 30 kDa cutoff centrifugal filter units (Amicon Ultra, Amersham, The Netherlands), and buffer exchange to PBS (10 mM NaxHyPO4, 137 mM NaCl, pH 7.4) was performed. Then, 4 µg of each sample was spotted on Amersham hybond N+ membranes (Cytiva, Marlborough, MA, USA) and dried. The staining was, on the one hand, with Azure A, which stains the glycans, and on the other hand, with a specific anti-HS antibody (Amsbio, Abingdon, UK). Before detection with anti-HS antibody, the membrane was blocked with 5% dry milk in 1x PBS with 0.05% Tween20 (PBST). This was then followed by an incubation with 0.5 µg/mL anti-HS antibody in 5% dry milk/PBST for 1 h at RT. The membrane was washed three times with PBST for 10 min at RT with shaking, and then the membrane was incubated with 0.8 µg/mL rabbit anti-mouse IgG + IgM HRP (Dianova, Hercules, CA, USA) for 1 h at RT and washed again three times. The chemiluminescent signal was recorded using Clarity™ Western ECL substrate (BioRad, Hercules, CA, USA).

### 4.7. Cell Culture Work

The used immune cells were prepared freshly from blood that was donated voluntarily and anonymously from various donors.

#### 4.7.1. PBMC (Monocyte) Isolation

Human whole blood was obtained from healthy female volunteers by venipuncture in K3EDTA vacuettes (Greiner bio-one, Kremsmünster, Austria). Following dilution of whole blood in HBSS without calcium and magnesium (ThermoFisher, Waltham, MA, USA), cells were layered over Ficoll Paque Plus (Cytiva, Marlborough, MA, USA) and separated using density centrifugation. Following the removal of the plasma layer, the buffy coat was aspirated and washed thrice with Hank’s balanced salt solution without magnesium and calcium. Cells were counted, and monocytes were assumed to be 1/6 of the total PBMC cell content.

#### 4.7.2. Preparation of Human Eosinophils

The blood was collected from healthy female Caucasian donors by means of venipuncture using K3EDTA vacuettes (Greiner bio-one, Kremsmünster, Austria). According to the manufacturer’s description, human eosinophils were isolated using EasySepTM direct eosinophil isolation kit (StemCell, Technologies, Vancouver, BC, Canada) and diluted to a final concentration of 1 × 10^6^ cells per mL in 1x PBS 1 mM EDTA.

#### 4.7.3. Chemotaxis

The ability of CCL2 and CCL26 to induce migration on monocytes and eosinophils was tested using a 48-well Boyden chamber system (Neuroprobe, Gaithersburg, MD, USA) with a polycarbonate, PVP (polyvinylpyrrolidone) membrane (25 × 80 mm, 5 µm pore size, Cytiva, Marlborough, MA, USA). Both CCL2 and CCL26 were recombinantly expressed in *E. coli* without tags and used at a concentration of 0.15 µM. A total of 50 µL of cell suspension (2 × 10^6^ cells/mL for monocytes and 1 × 10^6^ cells per mL for eosinophils per well) was added to the upper part of the chamber, and either chemoattractants or chemoattractants preincubated with 0.75 µM TNC or TNC alone were added to the lower well. All conditions and concentrations were measured in three independent wells in triplicates. After 2 h of migration at 37 °C and 5% CO_2_ (*v*/*v*), the membrane was removed, the site facing non-migrated cells was washed with HBSS−/−, and the whole membrane was air-dried. The migrated cells were fixed with Hemacolor fixing solution and stained with red and blue Hemacolor stain (Merck) in the case of PBMCs and with eosin (Merck) and blue Hemacolor stain (Merck) in the case of eosinophils. The chemotactic index (total amount of migrated cells per randomly migrated cells) was determined by taking five photos per well at 40 times magnification with a Nikon microscope. Chemotactic index (CI) was calculated as follows: the total number of cells migrated towards a particular chemokine concentration per number of randomly migrated cells towards buffer [91].

## Data Availability

There were no further new data created.
